# Knowledge, Attitudes and Practices (KAP) on Rift Valley Fever among Pastoralist Communities of Ijara District, North Eastern Kenya

**DOI:** 10.1371/journal.pntd.0004239

**Published:** 2015-11-13

**Authors:** Ismail H. Abdi, Hippolyte D. Affognon, Anthony K. Wanjoya, Washington Onyango-Ouma, Rosemary Sang

**Affiliations:** 1 Department of Statistics and Actuarial science, Jomo Kenyatta University of Agriculture and Technology, Nairobi, Kenya; 2 Social Science and Impact Assessment Unit, International Centre of Insect Physiology and Ecology, Nairobi, Kenya; 3 International Crops Research Institute for the Semi-Arid Tropics (ICRISAT), Bamako, Mali; 4 Institute of Anthropology, Gender and African Studies, University of Nairobi, Nairobi, Kenya; 5 Human Health Division, International Centre of Insect Physiology and Ecology, Nairobi, Kenya; University of Minnesota, UNITED STATES

## Abstract

Outbreaks of Rift Valley fever (RVF), a mosquito-borne viral zoonosis, have previously been associated with unusually heavy rainfall and extensive flooding. The disease is a serious public health problem in Africa and the Middle East, and is a potential global health threat. In Kenya, outbreaks of the disease have disproportionately affected impoverished pastoralist communities. This study sought to assess the knowledge, attitudes and practices (KAP) regarding RVF among the pastoralists of North Eastern Kenya, and to establish the determinants of KAP on RVF. A cross-sectional study involving 392 pastoralists living in Ijara district (Masalani and Ijara wards) was carried out using an interview questionnaire. All respondents interviewed (100%) had heard about RVF disease. They recognized that the disease is dangerous (99%), and had a positive attitude towards vaccination of animals (77%). However, few respondents knew that abortion (11%) and high mortality of young animals (10%) were key signs of RVF in animals. Very few (4%) use any form of protection when handling sick animals to avoid infection. Significant factors associated with knowledge were being in a household with a history of RVF infection (OR = 1.262, 95% CI = 1.099–1.447), having more livestock (OR = 1.285, 95% CI = 1.175–1.404) and the place of residence, Masalani (OR = 0.526, 95% CI = 0.480–0.576). Overall knowledge score on RVF was found to be a significant predictor of good preventive practice of the disease (OR = 1.073, 95% CI = 1.047–1.101). Despite the positive attitude that pastoralist communities have towards the prevention of RVF, there exist gaps in knowledge and good practices on the disease. Therefore there is need for public health education to address these gaps, and to identify and facilitate the removal of barriers to behavioural change related to the prevention of RVF.

## Author Summary

In this study, we showed that, although pastoralist communities living in Ijara district of Kenya had a positive attitude towards RVF prevention, they had little knowledge regarding the vectors, transmission, and signs or symptoms of RVF. Community members were not aware that abortion of pregnant animals and high death of young animals are alarming signs of RVF. A gap between the recommended and actual practices was also observed, as the necessary preventive measures against RVF were not being practiced. Poor practices regarding RVF prevention were associated with low knowledge regarding the disease; thus improving knowledge could lead to better practices among livestock keepers. To help address these gaps and promote good knowledge on RVF, we recommend that relevant institutions carry out public awareness campaigns. Radio may play an important role in conveying such health information to the public. There is also need to identify and facilitate removal of barriers to behavioural change related to the prevention of RVF among the study population. If effected, these interventions will therefore impact positively on pastoralist communities’ livelihoods, as disruptions of livestock market chains, which are a common occurrence whenever there is an outbreak of RVF, could become a thing of the past.

## Introduction

Rift Valley fever (RVF) is a mosquito-borne viral zoonosis that affects both domestic and wild animals [1]. The disease was first recognized and characterized in the Great Rift Valley of Kenya in 1931 during an investigation into an epizootic among sheep on a Naivasha farm [2]. The disease is caused by the RVF virus (RVFV), a member of the genus *Phlebovirus* in the family *Bunyaviridae* [3, 4]. Studies conducted in Kenya, Sudan and Saudi Arabia have shown a positive association between occurrence of RVF outbreaks and heavy rainfall, extensive flooding and increase of mosquito populations [5–11]. Such flooding leads to the hatching of a large number of flood water *Aedes* species, the reservoirs of the virus. When these mosquitoes lay eggs in flooded areas, transovarially infected adults may emerge and transmit RVFV to domestic animals close by [10]. The transmission of RVFV in domestic animals is either through bites of infected mosquitoes or by direct contact with infected animal tissues and bodily fluids particularly if associated with abortions [8].

Humans usually contract RVF through bites of infected mosquitoes; but, RVFV infection can also occur if they are exposed to blood, body fluids, or tissues of infected animals. Other documented risk factors include consuming animal products, particularly raw meat or milk, and sheltering livestock indoors in residential houses [12–14]. Both clinically and sub-clinically affected animals constitute a serious hazard to humans, as a source of infection. Currently, there is no evidence of person-to-person transmission of RVF infection in human beings. RVF causes flu-like symptoms that sometimes degenerate to severe disease, with encephalitis and haemorrhage that can result in 1–5% mortality [3, 11].

The disease is a serious public health problem in Africa and the Middle East. For instance, during the December 1997 RVF outbreak, 170 haemorrhagic fever-associated deaths were reported in Garissa, Kenya [14]. And with an estimated 27,500 human infections with the virus, the highest seroprevalence was found in Hulugho (32%) and Masalani (29%) divisions. This was the largest documented outbreak of RVFV infection in eastern Africa. Furthermore, the World Health Organization reported that the 2006/2007 RVF outbreak resulted in 684 reported cases in Kenya, including 155 deaths (a case-fatality rate of 23%), with nearly half (333) from North Eastern (NE) province [15]. Entomologic investigations on the epidemic revealed that in Garissa, peak abundance exceeded 5,000 mosquitoes per trap per night, and 72.3% of the mosquitoes were floodwater *Aedes*. *Aedes mcintoshi* and *Aedes ochraceus* were the *Aedes* species incriminated during this outbreak in Kenya. Of the 500 *Ae*. *mcintoshi* pools tested, 26 were positive for RVFV, and of the 450 *Ae*. *ochraceus* pools tested, 23 were positive for the virus. Although *Ae*. *ochraceus* is a known vector of the virus in West Africa, it was identified for the first time as a vector of RVFV in East Africa in the outbreak of 2006/2007. The detection of RVFV in significant proportions of these *Aedes* species, coupled with the observed high abundance, may have contributed to the transmission of the virus in the 2006/2007 outbreak in Kenya [16].

Since the first isolation of RVFV in Kenya in 1930, much attention has been paid to RVF virus, specifically on vaccines against the disease, on forecasting models of RVF, on monitoring and reporting of cases, and on incidences of death to the veterinary and public health authorities [17]. The socio-economic context within which RVF occurs remains a virtually neglected research area. Few studies have examined the wider socio-economic effects of the past outbreaks of RVFV [18]. During the RVF outbreak of 2006/2007, the pastoralist communities of North Eastern (NE) Kenya were specifically hard hit. In this region, livestock serve an important livelihood function for pastoralists with poor resilience to economic and environmental challenge [18, 19]. The total economic losses from livestock mortality in Garissa and Ijara districts were calculated at over Kshs 610 million (over US$9.3 million). Producers in Garissa district lost nearly 2.3 million litres in potential milk production. RVF- induced losses on the Kenyan economy were estimated to be over Kshs 2.1 billion (US$32 million) based on its negative impacts on agriculture and related sectors [19]. Periodic summary trade bans on livestock and livestock products from disease-endemic areas, have led to loss of livelihoods for many people [11, 20, 21].

Despite the importance of RVF, only a few studies assessing awareness of the communities regarding on disease have been conducted in Kenya [22]. A recent study on perceived risk factors has shown that the local community in Ijara does not consider poor handling of carcasses and aborted foetuses during RVF outbreak as important risk factors in the transmission of the disease [23]. A KAP (Knowledge, Attitude and Practice) survey is a representative study of a specific population to collect information on what is known, believed and acted on in relation to a particular topic [24]. Protection measures against a specific disease are related to the knowledge and beliefs of people hence KAP studies are increasingly becoming important in improving disease control activities [25]. Our intention in this study was to identify and contribute to filling the information gaps through assessing the knowledge, attitudes, and practices regarding RVF among the pastoralist community of Ijara district. In addition, we sought to establish key socio-economic and demographic determinants of KAP on the disease. The findings would provide information to government and other stakeholders to design effective and sustainable RVF prevention strategies in the study area. This would in turn impact positively on the livelihoods of pastoralist communities that are disrupted during RVF outbreaks, because they either depend on the income from sale of live animals, or of milk and milk products.

## Methods

### Description of the Study Area

**Fig 1:**
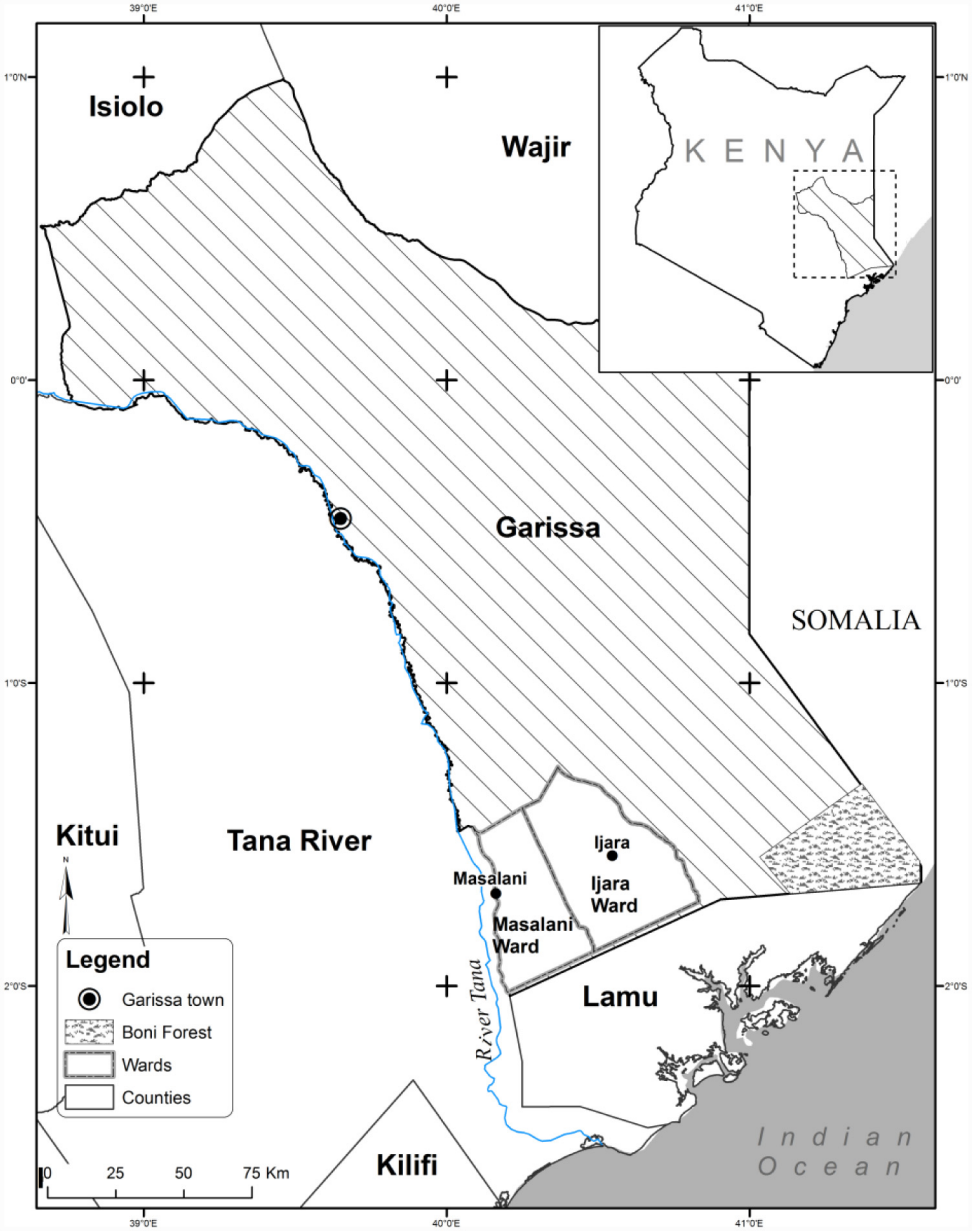
Map of the area where the study on knowledge, attitudes and practices (KAP) on Rift Valley fever among pastoralist communities in Kenya was conducted.

The study was carried out in Ijara district of North Eastern Kenya (Fig 1), which was one of the hotspots of RVF in previous outbreaks [26, 27]. The district is part of Garissa County and is subdivided into two administrative wards namely Masalani and Ijara. Data from the 2009 population and housing census of Kenya showed that Masalani had a population of 32,375 people in 4,755 households, with a population density of 21 people/sq km, while Ijara had a population of 11,474 people in 1,669 households, with a population density of 8 people/sq km [28]. About 90% of the inhabitants are predominantly ethnic Somali who depend on livestock for their livelihoods [22, 28].

The district borders Lamu County to the south, Tana River County to the west, Hulugho district to the east, and Fafi district to the north. The district is generally semi-arid, with high temperatures (ranging from 15° to 38°C) most of the year, and relatively cooler periods between April and August. The area receives bimodal rains ranging between 700 and 1000 mm. However, these are erratic and unreliable, making the district susceptible to droughts [29].

The district is characterized by black cotton soil and low undulating plains with low-lying altitude. The predominant vegetation in the area is shrubs of acacia species and grass; but the neighbouring Hulugho district has a big portion of coastal rainforest, commonly referred to as Boni forest. The main source of water supply is the Tana River [30].

### Study Design and Sampling Procedure

We used a cross-sectional study design to conduct the KAP survey -on RVF among pastoralists of Ijara district. Based on the formula for determination of sample size [31], 384 respondents were sufficient (with 95% confidence level, 5% margin of error and assuming a response distribution of 50%); however, we increased the sample size by 5% to account for non-response. Thus, 403 household heads were targeted for data collection. To address heterogeneity of the population, a stratified sampling design was used to select the participants in the two administrative wards for the study. Under this design, we used the proportional allocation to make the sample fraction constant for each stratum. This sampling design has a higher precision than simple random sampling [32]. Using a systematic random sampling method with a sampling interval of three (3) households, a total sample of 402 respondents consisting of 291 from Masalani (1°42’ S, 40°10’ E) and another 111 from Ijara (1°36’ S, 40°31’ E) was selected. The study population for the KAP survey consisted of the herd owners of Ijara district. The household head or other responsible person in the household aged at least 15 years was eligible to be interviewed. Only one person per household, whether male or female, was interviewed.

### Data Collection and Analysis

We obtained the necessary approval to conduct the study from the Ethical Review Committee of the Kenya Medical Research Institute (KEMRI) (Non-SSC protocol No. 316), to ensure adherence to Kenyan and international ethical guidelines (and regulations) governing research. We explained the purpose of the study to the research participants, local community and their leaders. During the data collection stage, participation in the study was voluntary, and all the respondents gave verbal consent. Parental consent and assent were also obtained prior to interview any minors. We ensured strict confidentiality in data handling and storage.

For two weeks in October 2013, data were collected using an interview questionnaire that was developed based on a literature review. Research experts at the International Centre of Insect Physiology and Ecology (*icipe*), and veterinarians at Kabete Veterinary Laboratory in Nairobi, Kenya reviewed the questionnaire; after which it was subjected to a pre-test on 10 households before administration to respondents in its final form. The pre-test was aimed at identifying any problems in the questionnaire, in order to eliminate them and to ensure adequate delivery of the required data. Enumerators fluent in the English and Somali languages were trained to administer the questionnaire. They asked the questions in Somali, and recorded the responses in English. We monitored the administration of the questionnaires daily, and checked the filled forms for the purpose of quality control.

The questionnaire used in the study consisted of five sections that included: (i) household characteristics and demography; (ii) household asset holding; (iii) knowledge about RVF vectors, symptoms, signs and transmission modes in humans and domestic animals; (iv) attitudes towards RVF and (v) prevention practices against RVF. In pastoralist societies, ownership of livestock is a proxy for wealth [33]; consequently, total tropical livestock units (TLUs) owned by each household was used as the wealth indicator. To quantify the different types of livestock owned by a household, the number of animals owned was converted to TLUs, where one TLU equals 250 kg of live weight (1 head of cattle = 0.7 TLU, 1 donkey = 0.5 TLU and 1 sheep or goat = 0.1 TLU) [34]. To obtain a value for this wealth indicator for each household, TLUs were multiplied by the number of animals for each species, and then summed up. In this study, households having a total TLU greater than the overall mean of the study population were considered wealthy while those below this were considered poor.

For each question in the knowledge section, a correct response was awarded one point while a wrong response or “don’t know” a zero point. A knowledge score for each participant was computed by summing the number of correct answers out of 25 questions. Attitudes towards the control and prevention of RVF was measured using six statements on a 5-point Likert scale (1 = strongly disagree; 2 = disagree; 3 = neither agree nor disagree; 4 = Agree; 5 = strongly agree) [35]. Those who agreed or strongly agreed to the statements were considered to have a positive attitude while the rest were considered to have a negative attitude. For the practices section, we asked eight yes/no questions to evaluate the precautionary measures the respondents took against RVF. We awarded respondents one point for each correct preventive measure mentioned, and a zero if the preventive measure was not mentioned. A practice score for each participant was computed by summing the number of correct responses out of the eight questions.

The variables in the data were coded for easy entry and analysis. Data were entered into Microsoft Excel 2007, cleaned to detect any missing or invalid variable and then imported to R software version 2.13.2 for analysis [36]. Results were presented in descriptive form using means, frequencies and proportions.

Using the knowledge score, we created a matrix where the first column is the number of correct responses (successes) and the second the number of incorrect responses (the failures). This matrix of “successes” and “failures” was used as the dependent variable [37] and then a regression analysis was performed using a generalized linear model of the binomial family with the logit link function to determine association between the response and explanatory variables. A similar matrix of number of “successes” and number of “failures” was created for the practice score and used as the dependent variable in the regression model. Multicollinearity between the covariates was assessed using the variance inflation factors (VIFs), where as a rule of thumb, a value of 10 or greater is a cause for concern [38]. Odd ratios were used to explain the result of significant factors. Hosmer-Lemeshow goodness-of-fit χ2 value was calculated for the model using the *Resource Selection* package of R to show whether or not the model predictors sufficiently describe the observed data. A p-value of less than 0.05 was considered significant for all statistical analyses.

## Results

### Characteristics of the Study Population

For the purpose of data analysis, we used only the completed questionnaires (392 out of 402 interviews). The whole sample of respondents was composed of 53.06% males and 46.94% females. Household heads formed 63.78% of the respondents, 28.83% were the wives of the household heads and the rest (7.39%) were a son, a daughter or other household member. Majority of the respondents (83.67%) were married, 4.59% were single, 8.67% were widowed and the rest (3.06%) were divorced. The average age of the respondents was 46.20 ± 13.94 years, and ranged from 16 to 85 years. There was no significant difference (p = 0.112) between the average age of respondents in Ijara (44.47 ± 13.30) and Masalani (46.88 ± 14.15), the two administrative wards where the survey was conducted. The average household size was 8.06 ± 2.89 with no significant difference (p = 0.755) between the average household size in Ijara (8.14 ± 3.14) and Masalani (8.03 ± 2.79).

Majority (87.76%) of the respondents had no formal education, and among those who attended school, the average years of schooling were 1.03 ± 3.11. Comparing the two study zones, no significant difference (p = 0.961) could be found between the average years of schooling of respondents in Ijara (1.02 ± 3.20) and Masalani (1.04 ± 3.08). However, majority of the households (88.01%) had at least one child in school. Regarding the ownership of communication gadgets, we found that almost all of the households (98.47%) owned and used mobile phones, with the average number of mobile phones being 2.45 ± 1.27 per household. As well, about 44.13% of the households own a radio.

Livestock types owned were cattle, sheep, goats and donkeys. The mean TLU was 27.39 ± 26.67. Among the households, the main source of income was the animals kept (61.48%), some businesses (19.13%), remittances (11.73%) that were generally from abroad and major cities in Kenya, and employment of a family member (7.65%).

All the respondents interviewed had heard about RVF disease. However, only 55.53% knew that mosquito is the vector of the disease in livestock. Relatives and friends was the common source of information about RVF (46.43%). About 9.69% of the households interviewed reported that they had a member of their household that had suffered from the disease. Among this group, about 63.15% reported to have lost a member of the household to the disease.

### Knowledge about Rift Valley Fever

The analysis of the knowledge score showed that out of a maximum of 25 points, the scores of the participants ranged from 1 to 19 with a mean of 9.62 ± 3.67 (equivalent to 38.50% of total score). Only 85 pastoralists (21.68% of the respondents) were able to get more than a half of the total scores, indicating low knowledge among community members.

When asked about how RVF manifests in animals, 9.95% and 10.46% of respondents mentioned high mortality of newborns and abortion, respectively, as signs of RVF in animals. Regarding the mode of transmission of RVF to humans, about 5.10% knew that touching an aborted foetus is a risk factor for RVF infection, while less than a half (46.68%) mentioned mosquito bites. Concerning knowledge of RVF signs in humans, majority of the respondents (91.84%) mentioned haemorrhage followed by high fever (63.27%). Table 1 shows the proportion of respondents who had knowledge on signs of RVF in man and livestock, as well as mode of transmission of RVF in humans.

**Table 1:** Knowledge about RVF among pastoralist communities in Ijara District, Kenya (n = 392).

	Frequency (n) *	Proportion (%)	95%Confidence Interval (%)
**Sign of RVF in animals** *			
High mortality of new borns	39	9.95	[6.97–12.93]
Sudden onset of abortion	41	10.46	[7.42–13.50]
Mucopurulent nasal discharge	64	16.33	[12.65–20.00]
Weakness	83	21.17	[17.11–25.24]
Profuse fetid diarrhoea	92	23.47	[19.25–27.68]
High fever	202	51.53	[46.56–56.50]
**Mode of transmission of RVF in humans** *			
Touching aborted foetus	20	5.10	[2.91–7.29]
Sheltering animals in house	32	8.16	[5.44–10.89]
Drinking raw milk	59	15.05	[11.50–18.61]
Touching body fluids	79	20.15	[16.16–24.14]
Eating raw/undercooked meat	147	37.50	[32.69–42.31]
Mosquito bite	183	46.68	[41.72–51.64]
**Sign of RVF in humans** *			
Haemorrhage	360	91.84	[89.11–94.56]
High fever	248	63.27	[58.47–68.06]
Headache	129	32.91	[28.24–37.58]
Muscle pain	74	18.88	[14.99–22.77]
Blurred vision	37	9.44	[6.53–12.35]
Backache	34	8.67	[5.88–11.47]

* Respondents in the study gave multiple responses for each sub-section

Logistic regression model was fitted to determine association between overall knowledge about RVF and participants’ characteristics (Table 2). The wealth status (measured in terms of TLU), place of residence (ward), and history of RVF infection by a member of the household were significantly associated with high knowledge. Households with a history of RVF infection were more likely to have good knowledge (OR = 1.262, 95% CI = 1.099–1.449, p = 0.001) compared with those who had no history of the disease. Residents of Masalani ward were less likely to have good knowledge compared to their counterparts in Ijara (OR = 0.526, 95% CI = 0.480–0.576, p = 0.001). Being in a wealthy household increased the odds of a participant having good knowledge (OR = 1.284, 95% CI = 1.175–1.404, p = 0.001).

**Table 2:** Multiple logistic regression analysis of the association between factors and good knowledge about RVF.

Variable	Category	OR	95% CI	P-value
Place of residence	Masalani ward	0.526	0.480–0.576	0.000
	Ijara ward (reference)	1		
Gender	Male	1.060	0.969–1.160	0.204
	Female (reference)	1		
Education	Has formal education	0.970	0.846–1.111	0.663
	No formal education (reference)	1		
TLU	Wealthy	1.284	1.175–1.404	0.000
	Poor (reference)	1		
Married status	Married	0.987	0.878–1.110	0.825
	Not married (reference)	1		
Household member Previously infected with RVF	Yes	1.262	1.099–1.449	0.001
	No (reference)	1		
Age		1.001	0.998–1.005	0.371
Household size		0.995	0.980–1.010	0.483

OR = odd ratio, CI = confidence interval.

All the independent variables had VIFs less than 1.2 indicating absence of serious multicollinearity. The Hosmer-Lemeshow goodness-of-fit test yielded a chi-square value of 0.672 on 8 degrees of freedom, and a p-value of 0.99 suggesting that the model fits the data well.

### Attitude of Respondents towards the Prevention and Control of RVF

**Fig 2:**
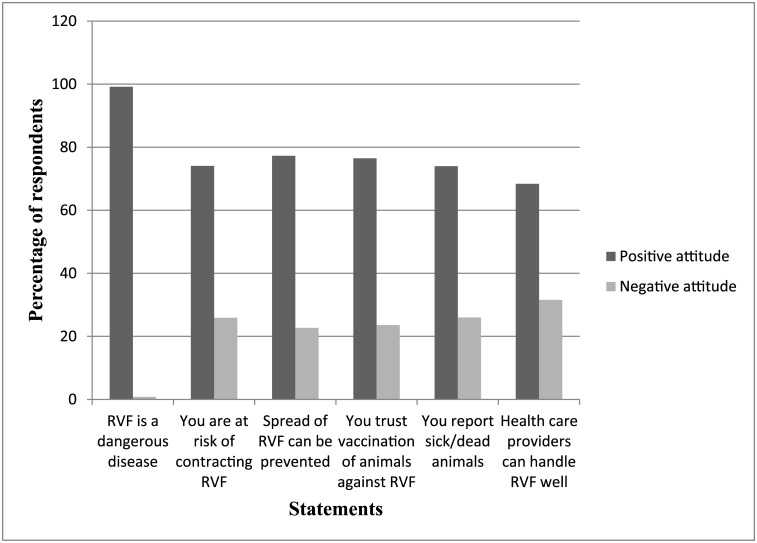
Attitude of respondents regarding RVF prevention and control.

In all the six statements used to measure attitude, a high percentage of respondents had a positive attitude towards the prevention and control of RVF (Fig 2). There was no significant difference in attitude between male and female respondents; RVF is a dangerous disease (p = 0.492), you are at a risk of RVF infection (p = 0.796), spread of RVF from animals to humans can be prevented (p = 0.957), you trust vaccination of animals against RVF protects them against RVF infection (p = 0.845), you will report sick or dead animals to the local authorities/veterinary officers (p = 0.624), and health care providers can handle RVF outbreaks very well (p = 0.360).

Comparing the two areas of residence, there was no significant difference between the attitudes of respondents in the statements “You are at risk of contracting RVF” (p = 0.069) and “You trust vaccination of animals against RVF that protects them against RVF infection” (p = 0.116).

### Practices Regarding Rift Valley Fever Prevention

When asked about preventive measures against RVF, the least mentioned were use of any form of protection when handling sick animals (4.34%), avoiding touching an aborted foetus (5.61%), and avoiding sheltering animals near the house (9.95%). Drinking of raw milk and contact with body fluids from animals was avoided by 17.09% and 20.66% of the respondents respectively. About two-fifths of the respondents used various ways of reducing mosquito bites, and 42.35% avoided slaughtering their sick animals for meat. However, majority of the respondents (95.41%) cooked meat thoroughly to avoid RVF infection.

Regarding association of participants’ characteristics with good practice, the fitted logistic regression model (Table 3) did not show any significant factor among all the socio-demographic variables. The knowledge score (knowledge about RVF) of the participants had significant positive association with good practice (OR = 1.073, 95% CI = 1.047–1.101, p = 0.000).

**Table 3:** Multiple logistic regression analysis of the association between factors and good preventive practices against RVF.

Variable	Category	OR	95% CI	P-value
Place of residence	Masalani ward	1.198	0.981–1.468	0.075
	Ijara ward(reference)	1		
Gender	Male	0.969	0.819–1.148	0.719
	Female(reference)	1		
Education	Has formal educ	1.131	0.876–1.454	0.340
	No formal educ (reference)	1		
TLU	Wealthy	1.047	0.882–1.243	0.596
	Poor (reference)	1.		
Married status	Married	1.240	0.992–1.558	0.062
	Not married (reference)	1		
Previous infection by HH member	Yes	0.999	0.767–1.293	0.994
	No (reference)	1		
Age		1.004	0.998–1.010	0.164
Household size		0.986	0.958–1.014	0.331
Knowledge score		0.073	1.046–1.101	0.000

OR = odd ratio, CI = confidence interval.

All the independent variables had variance inflation factors (VIF) less than 1.4 indicating absence of serious multicollinearity. The Hosmer-Lemeshow goodness-of-fit test yielded a chisquare value of 1.106 on 8 degrees of freedom and a p-value of 0.99, suggesting a good fit.

## Discussion

Everyone interviewed had heard about RVF, but this was not strange, as Ijara was one of the districts seriously affected by the disease in the outbreaks of 1997/1998 and 2006/2007 in Kenya [26, 27]. Despite this general awareness, there was an indication of low level of knowledge based on overall scores. Regarding RVF signs in animals (Table 1), apart from high fever, other signs in animals were mentioned by less than 25% of the respondents. This finding was consistent with a study done among livestock keepers of Tanzania that indicated that there was little knowledge on RVF (all clinical signs scored less than 50%) [39]. However, this finding seems to run contrary to a comparative assessment of the 2006/2007 RVF outbreak in Kenya and Tanzania. In the assessment, it was revealed that the Somali pastoralists in Kenya could provide more accurate and detailed clinical descriptions of diseases affecting their livestock, including RVF, than Maasai pastoralists of Tanzania [22]. This was probably due to the educational messages conveyed during the RVF outbreak.

The low level of knowledge shown in our study could be due to community members forgetting a good deal of information about the disease, considering that the survey was carried out almost seven years after the last outbreak. This is an indication that continued health education is necessary, and should be intensified when there is increased risk of an outbreak, or when an outbreak alert has been issued.

Regarding the symptoms of RVF in human beings (Table 1), majority of the community members in this study identified haemorrhage as a key sign of the disease, followed by high fever. It seemed the most memorable aspect of the disease is its hemorrhagic manifestation in humans. Although a large proportion of RVF cases present with mild to unrecognized illness, haemorrhage, which presents in a small proportion of those who get infected, is associated with severe illness. Haemorrhage is what most people remember about this severe disease. In fact, Somalis call RVF *san-dig*, which means “bloody nose” [22]. The use of this term in this survey could explain the high proportion of respondents mentioning hemorrhage as a sign of RVF in humans.

Most of the respondents were interested to know more about the disease, and there was a high positive attitude regarding RVF prevention (Fig 2). This may be an indication that the pastoralist community of Ijara district can easily accept, and adapt educational programmes that aim at RVF prevention and control. A relatively smaller proportion of the respondents agree to reporting sick or dead animals to the relevant authorities. This lack of reporting makes it difficult for veterinary services to appreciate the scale of the problem when an outbreak occurs in order to take appropriate steps to prevent further transmission. There is need to sensitize the community on the importance of reporting abortion storms and cases of deaths in animals. Hassan *et al*. [5] demonstrate that, collaboration between various stakeholders is an important element in the strategy for the prevention and control of RVF.

Understanding the risk factors for RVF infection is crucial to implementation of prevention measures and decreasing the probability of infection during an outbreak. The low knowledge of respondents on the role of mosquitoes in the transmission of the disease is of particular concern. The results of this study (Table 1) show that less than one- half of community members knew RVF in human beings is contracted through mosquito bites, touching an aborted foetus and the use of raw animal products. Community members in the study area did not practice preventive measures against these risk factors. A recent study on perceived risk factors shows that the local community in Ijara district does not consider poor handling of aborted foetuses during an RVF outbreak as an important risk factor in the transmission of the disease [23]. The high consumption of raw milk reported in this study compares well to a study among herdsmen and their families in Ghana where only 35.3% of the respondents indicated that they always boiled the milk before consumption [40]. Findings from a study in Tanzania also reveal that pastoralist communities practice sheltering of animals in their houses and drinking of raw milk, despite local authorities prohibiting them from doing so [39]. These unsafe practices increase the vulnerability of the pastoralist communities and their livestock to not only RVF, but also other zoonotic diseases. The culture and beliefs of the community could explain the existence of practices such as consumption of raw milk and use of livestock in sacrificial rituals [17]. There is need for health education to encourage behavioural change, and eliminate these unsafe practices in the study area.

This study showed that overall knowledge on RVF is not associated with most socio-demographic variables like age, sex, education level, marital status, and household size. Residents of Masalani ward had lower knowledge as compared to Ijara residents. Masalani is an urban area and residents do not keep their livestock at home. This may have led to less experience in taking care of livestock, and concomitant low knowledge on RVF. Households that had a history of RVF infection through a family member had better knowledge, probably explained by the first hand experience in dealing with the sickness (or unfortunate death) in the family. Households with more livestock exhibited higher knowledge of the disease compared to those with fewer animals. Families with larger herds may have had more experience of the disease in their animals, and had to manage the situation in their livestock as shown in the case of cattle trypanosomiasis in West Africa [41].

Majority of the livestock keeping community of Ijara district have no formal education and therefore, reading materials such as posters may not be suitable for them. It is, however, important to note that almost all the households have school going children who could facilitate interpretation of the poster information, and may be used as agents in educating their parents. To provide information about RVF directly to the herd owners and increase awareness of the community members, radio programmes in vernacular languages could be of help. Community members can use the communication gadgets they already have, such as radios and mobile phones, to acquire information on RVF. The use of radio as an efficient media of dissemination of information to educate livestock keepers on RVF has been recommended in Kenya [42] and is a widely used communication medium in NE Kenya [43]. A high number of the respondents in this study reported having obtained information about RVF from informal channels such as relatives and friends. There is need to organize and use community meetings (*barazas*) as a means to reach those who may not get information regarding RVF through formal channels such as the media, schools and health centres.

Good practices regarding prevention of RVF was found to be significantly associated with high knowledge on the disease. This finding compares well with a study where knowledge on dengue fever was found to be the only predictor of good practice of preventing the disease in Cuba [44]. In another study carried out in Sudan, it was also shown that poor knowledge about malaria was a significant factor for death from malaria among the household members [45]. Improving knowledge through public education campaigns could, therefore, lead to better practices among livestock keepers.

### Conclusions and recommendations

We found a low level of good knowledge on RVF among the respondents based on the overall scores. Despite this low level of knowledge, they had a positive attitude towards RVF control and prevention. Community members do not practice recommended preventive measures against the disease. Therefore, there is need for massive awareness programmes to raise the knowledge of community members. Health education needs to be continuous and should be intensified when there is increased risk of an outbreak or when an outbreak alert has been issued. This could go a long way towards community members adopting effective measures for preventing RVF infection and in better controlling outbreaks. Use of radio for broadcasting messages on RVF and the development of information, education and communication (IEC) programmes may help achieve improved knowledge on the disease.

Awareness messages should focus on informing people on vectors of RVF, signs of the disease in animals, mode of transmission of RVFV in humans, as well as the importance of reporting suspected cases to relevant authorities. Messages that emphasize preventive measures against the disease such as “Avoid mosquito bites”, “Avoid contact with aborted foetus”, “Do not slaughter sick animals”, “Do not shelter animals in the house” and “Avoid drinking raw milk” may be used. We recommend that future studies on RVF should identify and facilitate removal of barriers to behavioural change related to the prevention of RVF among the population.

## Supporting Information

S1 Checklist Checklist of items included in reports of Cross-sectional studies. (DOC)

S1 Data Knowledge, Attitude, and Practices data collected in October 2013 in Ijara district of North Eastern Kenya. (XLSX)
